# Filariasis among Australian Troops

**Published:** 1919

**Authors:** 


***Filariasis Among Australian Troops.*** By R. RIMMER. *British Medical Journal,* October 12, 1918.

The first two cases admitted were diagnosed filaria. In the one case there was a well marked lymph scrotum with an acute attack of lymphangitis at the time of admission. The local condition improved with hot fomentation, purgation, and rest in bed. The second case was admitted with hematochyluria and some pain and tenderness over the bladder. In neither of these cases were any embryos found in the blood stream, although repeated examinations were made.

The third case was admitted with a diagnosis of nephritis. There was no pain, tenderness, or swelling. The urine was typically chylous. The obstruction seemed to be purely lymphatic. In this case the embryos were fairly numerous in the peripheral circulation. After 9.30 P. M. two or three were present in each small drop, the thin film method of examination being employed.

The fourth case showed only enlarged glands in both groins, most marked on the right; at intervals they became tender and more swollen. The enlarged glands, which were at first thought to be syphilitic, were present in both groins and extended from the interior superior spine of the ilium to the pelvis. All glands were discrete, and no thickened lymphatic cords could be felt. Examination of the blood at midnight showed the presence of the embryos.

The four men had all lived in middle and and southern Queensland for considerable periods.

It would be interesting to know if there are many cases of filarial infection among Australian troops who have lived in Queensland.

